# Study protocol for a randomized trial on timely delivery versus expectant management in late preterm small for gestational age pregnancies with an abnormal umbilicocerebral ratio (UCR): the DRIGITAT study

**DOI:** 10.1186/s13063-022-06561-w

**Published:** 2022-08-01

**Authors:** M. Smies, S. E. Damhuis, R. G. Duijnhoven, A. G. Leemhuis, S. J. Gordijn, W. Ganzevoort

**Affiliations:** 1 Department of Obstetrics and Gynaecology, Amsterdam University Medical Centers, University of Amsterdam, Amsterdam, The Netherlands; 2 Department of Obstetrics and Gynaecology, University Medical Center Groningen, University of Groningen, Groningen, The Netherlands; 3 Department of Neonatology, Amsterdam University Medical Centers, University of Amsterdam, Amsterdam, The Netherlands

**Keywords:** Fetal growth restriction, FGR, Third trimester, Small for gestational age, SGA, Umbilicocerebral ratio, UCR, Cerebroplacental ratio, CPR, RCT trial, Placental insufficiency

## Abstract

**Background:**

The clinical inability to correctly identify late fetal growth restriction (FGR) within a group of fetuses who are identified as small for gestational age (SGA) is an everyday problem for all obstetrician-gynecologists. This leads to substantial overtreatment of healthy small fetuses but also inadequate detection of the growth-restricted fetuses that may benefit from timely delivery. Redistribution of the fetal circulation, signaled by an abnormal ratio of the Doppler velocity flow profiles of the umbilical artery and the middle cerebral artery, more specifically an increased umbilicocerebral ratio (UCR) (or its inverse: a decreased cerebroplacental ratio (CPR)), is an adaptation to chronic hypoxemia and nutritional scarcity with long-term consequences in survivors. The relevance of an abnormal UCR has been signaled extensively, and there is a general consensus that it is a signal of FGR, independent of size, with a strong association with poor outcomes. Yet, in the current literature, no comparisons of a monitoring-delivery strategy based on unfavorable UCR have been published. The objective of the Doppler Ratio In fetal Growth restriction Intervention Trial At (near) Term (DRIGITAT) is to evaluate if the timing of the delivery based on an abnormal UCR in late preterm fetuses identified as SGA improves neurodevelopmental outcomes at 2 years of age.

**Methods:**

The DRIGITAT study is a national multicenter cohort study of women with singleton pregnancies between 32 and 37 weeks of gestation identified as SGA, with a nested randomized controlled trial (RCT) in case of an abnormal UCR (> 0.8). Recruiting centers are in The Netherlands. In the nested RCT, women are randomized to either immediate induction of labor or expectant management from 34 weeks in case of severely abnormal size (EFW or FAC < p3) and from 36 weeks in case of mildly abnormal size (EFW or FAC p3–p10). The primary outcome measure is the 7-point average difference in the composite cognitive score (CCS) and composite motor score (CMS) on the Bayley-3 at 2 years. Secondary outcome measures include a composite outcome of neonatal morbidity, perinatal mortality, mode of delivery, maternal quality of life, costs, and predictive value of serum biomarkers. Analyses will be by intention to treat. The required sample size is determined for the nested RCT as 185 patients.

**Discussion:**

This study will provide insight into the diagnostic efficacy of UCR measurement in the evaluation of SGA fetuses in order to differentiate the healthy SGA fetus from the growth-restricted fetus and to determine if a fetus with abnormal UCR benefits from early delivery.

**Trial registration:**

Healthcare Evaluation Netherlands NTR6663. Registered on 14 August 2017.

## Administrative information

Note: The numbers in curly brackets in this protocol refer to SPIRIT checklist item numbers. The order of the items has been modified to group similar items (see http://www.equator-network.org/reporting-guidelines/spirit-2013-statement-defining-standard-protocol-items-for-clinical-trials/).

**Table:** 

Title {1}	Study protocol for a randomized trial on timely delivery versus expectant management in late preterm small for gestational age (SGA) pregnancies with an abnormal umbilicocerebral ratio (UCR): the DRIGITAT study
Trial registration {2a and 2b}.	Trial NL6475 (NTR6663), registered 14/08/2017
Protocol version {3}	Version 8.0 dated July 13, 2020
Funding {4}	ZonMw, reference number 843002825
Author details {5a}	^1^ Department of Obstetrics and Gynaecology, Amsterdam University Medical Centers, University of Amsterdam, Amsterdam, The Netherlands; ^2^ Department of Obstetrics and Gynaecology, University Medical Center Groningen, University of Groningen, Groningen, The Netherlands; ^3^ Author formerly known as van Wassenaer-Leemhuis. Department of Neonatology, Amsterdam University Medical Centers, University of Amsterdam, Amsterdam, The Netherlands;
Name and contact information for the trial sponsor {5b}	Dr. J.W. Ganzevoort Email: j.w.ganzevoort@amsterdamumc.nl Amsterdam University Medical Centres, location AMC PO box 22660 | 1100 DD Amsterdam, the Netherlands Telephone: +31 20 5663769
Role of sponsor {5c}	The sponsor has the final role in the study design; collection, management, analysis, and interpretation of the data; writing of the report; and the decision to submit the report for publication and will have ultimate authority over any of these activities. The funding agency has provided funds to perform the study according to the predefined plan. It has no role or authority in any of these activities, under the assumption that fundamental changes to the protocol need to be approved.

## Introduction

### Background and rationale {6a}

Fetal growth restriction (FGR) is traditionally defined as small for gestational age (SGA), a definition based on size, usually below the 10th percentile on growth reference centiles, and thus by definition affects 10% of all fetuses. SGA however indicates a reference-based *small-sized* fetus, and FGR indicates a fetus smaller than its intrinsic growth capacity, which may not be below the 10th percentile on the reference curve [1]. Among SGA fetuses is a considerable group of fetuses that is constitutionally small but healthy and among the appropriate for gestational age (AGA) fetuses is a group of fetuses that are growth restricted despite an apparently normal weight.

The pathophysiological mechanism in FGR is often uteroplacental insufficiency, with multiple underlying causes, leading to the failure of the placental exchange unit to serve fetal needs. When the growth-restricted fetus remains undelivered, the insufficiency progresses and the prolonged placental restraints put the fetus at risk for fetal demise [2]. Also, while remaining in utero, permanent alterations in fetal physiology increases the fetus’ risks of disease in adulthood [3]. When delivered timely, usually in the late preterm period, the baby is at risk for neonatal transitional disease and gross morbidity.

Because of the diagnostic substitution of SGA with FGR, the effect of any approach is diluted by the inability to identify fetuses with true placental growth restriction that may benefit from timely interventions by avoiding fetal risks that surpass the neonatal disadvantages [4]. A major challenge is to differentiate the FGR fetus from the healthy fetus within the group of SGA fetuses. The other challenge, outside the scope of this trial, is to identify the growth-restricted fetus within the group of apparent normal size fetuses.

Functional parameters, such as Doppler ultrasound and serum biomarkers, can help distinguish the FGR fetuses from healthy SGA fetuses. Redistribution of the fetal circulation, signaled by an increased umbilicocerebral ratio (UCR) or its inverse a reduced cerebroplacental ratio (CPR), caused by a decrease in resistance in the middle cerebral artery (MCA, reflecting cerebral flow) and an increased resistance in the umbilical artery (UmbA, reflecting placental flow), is an adaptation to scarcity with long-term adverse consequences in survivors [5–11]. Serum biomarkers, including soluble fms-like tyrosine kinase-1 (sFlt-1) and placental growth factor (PlGF), have received attention as markers for placental function [12, 13], as they have a considerable association with relevant outcomes [14, 15].

There are also clear associations of late prematurity, a possible consequence of timed delivery, with significant adverse neurodevelopmental outcomes. Even in the absence of severe neonatal morbidity, which is uncommon in late prematurity, there are significant effects from preterm delivery, predominantly transitional disease (neonatal jaundice, hypoglycemia) that are temporary but may have a bearing on long-term neurodevelopmental outcomes [16]. These may be related to a simple effect from gestational age, but are more likely due to the underlying reason for preterm delivery.

The dilemma is obvious: previous studies clearly show some diagnostic accuracy of the UCR resulting in many (doctors) to believe in an “obvious” effective test-treatment combination in SGA fetuses [11, 17]. Intuitively, physicians balance the effect on outcomes from cohort evidence of associations of the diagnostic tools with the cohort evidence of the effect of gestational age. In this process, the fear of the worst outcome (stillbirth), despite its low incidence, often drives decisions towards early interventions with a high risk of mild morbidity, also for the long term. The above leads to practice variation due to different perceptions of risk. However, prospective comparative evidence is lacking. There is international consensus that a RCT on intervention on abnormal UCR is now opportune, including the investigation of serum biomarkers for their potential added value in guiding timing of delivery.

We hypothesize that with the addition of the UCR measurement in evaluation of SGA fetuses, we are better able to differentiate the SGA fetus at risk from the healthy SGA fetus. Furthermore, by directing intervention towards timely delivery based on an abnormal UCR, we hypothesize that (1) a higher number of growth-restricted fetuses are no longer exposed to prolonged risks of placental insufficiency and are delivered timely with better neurodevelopmental outcomes and (2) less fetuses that are SGA and physiologically small are subjected to unnecessary interventions; all the above leading to improved health outcomes and saved costs.

### Objectives {7}

The objective of this study is to use the diagnostic efficacy of the UCR in pregnant women with fetuses identified as SGA to differentiate those fetuses who are subject to risks related to placental insufficiency and thus growth restricted and those fetuses who are not at increased risk and thus healthy. Subsequently, this identification will be used for time delivery.

The research aims of the DRIGITAT study are as follows:

1. To investigate if, in a cohort of late preterm SGA fetuses, timely delivery in a nested RCT in case of an abnormal UCR improves immediate perinatal outcome and long-term neurodevelopmental outcome as tested with Bayley-3 at 2 years (hypothesis testing)
2. To assess the predictive value of serum biomarkers, for the primary outcome and other main secondary outcomes (hypothesis generating)
3. To estimate the costs and cost-effectiveness of the above monitoring intervention strategy, with potentially shifting the majority of fetuses to more expectant management and a minority towards earlier induction of delivery

### Trial design {8}

The trial design is a cohort study of SGA pregnancies with a nested randomized controlled trial in fetuses with an abnormal UCR, superiority design.

## Methods: participants, interventions, and outcomes

### Study setting {9}

The DRIGITAT is a nationwide trial conducted in secondary and tertiary care hospitals in The Netherlands that evaluate and manage (late) fetal growth restriction. The trial is embedded in the Dutch Obstetric Consortium, a collaboration of obstetrics hospitals in The Netherlands. A list of current study sites can be obtained at www.zorgevaluatienederland.nl/DRIGITAT.

### Eligibility criteria {10}

In order to be eligible for inclusion in the observational cohort, a patient must meet all of the following criteria:

- Singleton pregnancy
- Gestational age from 32 + 0 up to and 36 + 6 weeks
- Identified SGA (estimated fetal weight (EFW) or fetal abdominal circumference (FAC) below the 10th percentile)

In order to be eligible to participate in the nested RCT, a patient must meet all of the above-described criteria and additional following criteria:

- Abnormal UCR of more than 0.8 (equals a CPR of less than 1.25) on at least 2 occasions with an interval of at least 1 workday (minimum of 15 h)

Two groups can be included in the RCT, dependent on FGR severity.

1. EFW and/or FAC < p3 AND gestational age from 34 + 0 up to and including 36 + 6 weeks of gestation
2. EFW and/or FAC < p10 AND gestational age from 36 + 0 up to and including 36 + 6 weeks of gestation

The EFW for inclusion or randomization should be calculated with the Hadlock 3 formula (including the FAC, head circumference (HC) and femur length (FL) [18], and the percentile value of the EFW should be based on the Hadlock reference curve [19].

The percentile value of the FAC should be based on the Verburg reference curve [20].

#### Exclusion criteria

A potential patient who meets any of the following criteria will be excluded from participation in this study (both the cohort and RCT):

- Maternal age < 18 years
- Inability to give informed consent (lack of comprehension, language)
- Uncertainty about the estimated due date
- Suspicion of congenital anomalies which can influence the prognosis of the pregnancy or health of the fetus
- Proven chromosomal abnormalities
- Maternal or fetal indication for short-term delivery

### Who will take informed consent? {26a}

A trained and authorized member of de local study team will counsel the patient and take informed consent.

### Additional consent provisions for collection and use of participant data and biological specimens {26b}

Additional consent will be obtained to store residual material in a biobank for 50 years. This concerns the remaining blood after biomarker analysis of the maternal blood sample that is taken from all participants. This consent can also be used to store placental tissue or other residual material collected during standard care. Consent is also obtained to contact participants for additional follow-up investigation that does not fall within the initial research question of this study.

### Interventions

#### Explanation for the choice of comparators {6b}

Concerning the hypothesis that a healthy SGA fetus has normal Doppler measurements and the FGR fetus has abnormal Doppler measurements as a reflection of redistribution to compensate for hypoxia by prioritizing the brain for oxygenated blood, the best solution would be to end the unfavorable intra uterine condition and to deliver the fetus with an insufficient placenta. The disadvantage is relative preterm birth that not only ends starvation and hypoxia but also ends maturation of the fetus. When the growth-restricted fetus remains undelivered, the progressive insufficiency puts the fetus at risk for fetal demise. Also, while remaining in utero, permanent alterations in fetal physiology increases the fetus’ chances of disease in adulthood. On the other hand, when delivered timely, usually in the late preterm period, the baby is at risk for neonatal transitional disease and gross morbidity. When a small fetus has normal Dopplers it is considered to be a healthy SGA fetus and delivery can be awaited.

#### Intervention description {11a}

Delivery from 34 weeks onwards when UCR is abnormal and fetal size is severely abnormal (EFW or FAC below p3), and delivery from 36 weeks when UCR is abnormal and fetal size is mildly abnormal (EFW or FAC p3–p10). Delivery has to be pursued as soon as possible from the moment of randomization.

#### Criteria for discontinuing or modifying allocated interventions {11b}

Safety criteria have been established for discontinuing the allocated intervention in case of expectant management. Guidance and safety criteria are given regarding the timing of the delivery in the cohort and in the expectant arm of the RCT. Delivery is indicated when:

1. Cardiotocography (CTG) suggests fetal distress
2. Doppler ultrasound of the umbilical artery suggests very high antenatal risks:
  - Reversed end-diastolic flow (REDF) in the UmbA on two occasions with at least 1 day in between from a gestational age of more than 32 weeks
  - Absent end-diastolic flow (AEDF) in the UmbA on two occasions with at least 1 day in between from a gestational age of more than 34 weeks
  - Pulsatility index (PI) in the UmbA above the 95th percentile on two occasions with at least one day in between from a gestational age of more than 37 weeks
3. EFW or FAC below the 10th percentile from a gestational age of 38 weeks in the expectant arm of the RCT or refusal to participate in the RCT
4. EFW or FAC below the 3rd percentile from a gestational age of 38 weeks in the cohort
5. EFW or FAC below the 10th percentile from a gestational age of 40 weeks in the cohort
6. A non-reassuring CTG trace
7. Presence of other clinical signs indicating short-term delivery according to standard protocol, such as severe pre-eclampsia or repeated episodes of reduced fetal movements

In case of allocation to immediate delivery, there are no criteria for discontinuing the intervention other than participant request. When a woman is not willing to be randomized for the timing of the delivery, she will be followed up for intention-to-treat analysis and will receive usual care and timing of delivery.

#### Strategies to improve adherence to interventions {11c}

Standard phrases to use in the electric patient files for the guidance of monitoring and management have been made available. The research team is approachable 24/7 for any relevant questions. The adherence to the allocated intervention is monitored by the study monitor through the electronic database where time and indication of delivery are recorded in the case report form.

#### Relevant concomitant care permitted or prohibited during the trial {11d}

The DRIGITAT protocol allows centers to provide their usual care according to local protocol. All other care deemed clinically necessary is allowed. The minimum required procedures and assessments that participants must undergo are described below. Most of these procedures and assessments are already embedded in local protocols on FGR of participating hospitals.

1. Cardiotocography: CTG monitoring takes place in all fetuses with an abnormal UCR with a frequency as indicated by local protocol. The study protocol advises CTG monitoring at least twice a week in case of abnormal UCR.
2. Ultrasound investigation: the study protocol recommends to perform a weekly ultrasound scan on all participants. The ultrasounds will be performed by trained personnel.
  - Fetal biometry: routine fetal biometry measurements according to the departmental protocol should take place every 10–14 days.
  - Fetal and maternal Doppler measurements: fetal Doppler measurements should be done on a weekly basis. At each visit, multiple Doppler measurements are taken, and the measurement of best quality is used. This is usually the PI of the lowest value. When the UCR is > 0.8 for the first time, Doppler measurements should be repeated to confirm the abnormal UCR. Ideally, the measurement is repeated the next day, with a minimum interval of 15 h. Randomization can take place when the UCR is abnormal on two different occasions with at least one day (a minimum of 15 h) in between.
    Middle cerebral artery (MCA) Doppler measurement: Doppler blood flow waveforms should be measured in the middle cerebral artery just past the level of the bifurcation of the internal carotid artery [21].
    Umbilical artery (UmbA) Doppler measurement: Doppler blood flow velocity waveforms should be measured in the umbilical artery in a free-floating loop of the mid-section of the umbilical cord [22].
    Uterine artery (UtA): Doppler blood flow waveforms should be measured in the uterine artery, ideally 1 cm downstream from the crossover point with the external iliac artery [23].
  - Amniotic fluid measurements: amniotic fluid index (AFI) or the single deepest vertical amniotic fluid pocket (SDP) should be measured.
3. Maternal surveillance: standard maternal surveillance will include, at least, the measurement of blood pressure and, if abnormal, a check for proteinuria.
4. Placental pathology: we advise all hospitals to send the placenta to their own pathology department for histopathologic investigation according to national consensus protocol [24].

#### Provisions for post-trial care {30}

The sponsor has a liability insurance which is in accordance with article 7 of the Dutch medical research involving human subjects act (WMO). The sponsor (also) has an insurance which is in accordance with the legal requirements in the Netherlands (Article 7 WMO and the Measure regarding Compulsory Insurance for Clinical Research in Humans of 23 June 2003). This insurance provides cover for harm to participants through injury or death caused by the study. This insurance applies to harm that becomes apparent during the study or within 4 years after the end of the study.

### Outcomes {12}

#### Primary outcome

The primary outcome includes neurodevelopmental outcomes at 2 years of age in all children born in the RCT and in a subset of children born in the cohort. The neurodevelopmental score will be assessed by means of the Bayley-III test [25].

The Bayley Scales of Infant and Toddler Development (Bayley-III is the current version) is a standard series of measurements to assess the development of infants and toddlers, ages 1–42 months. This measure consists of a series of developmental play tasks and takes between 45 and 60 min to administer and derives a developmental quotient (DQ) rather than an intelligence quotient (IQ). Raw scores of successfully completed items are converted to scale scores and to composite scores.

The developmental score is derived separately for the cognitive and motor domain with a mean of 100 and SD of 15. Higher scores indicate a better level of development. A 7-point difference (− 0.5 SD) between the two treatment groups in this trial is considered to represent a meaningful difference. Women who were eligible for the RCT but did not want to be randomized for intervention will also be asked to participate in the follow-up with Bayley-III testing.

#### Secondary outcomes

In all participants (RCT and cohort), the following outcomes will be analyzed:

- Short-term perinatal morbidity, defined as a composite outcome of level of neonatal care, intravenous antibiotics treatment more than 3 days, any respiratory support, any cerebral damage, cranial ultrasound, neonatal jaundice, neonatal hypoglycemia, and its individual components. These are relatively mild outcomes since neonatal morbidity in near-term deliveries is uncommon, while differences in primary outcomes are likely significant.
- Perinatal mortality.
- Mode of delivery.
- Maternal quality of life through the European Quality of life 5-Dimension 5-Level (EQ5D-5L) Questionnaire, 6 weeks, 12 months, and 24 months after delivery.
- Pediatric development using the Ages & Stages Questionnaire, Third Edition (ASQ-3) and the Child Behavioural Checklist for ages 1.5–5 (CBCL/1.5-5) 24 months after delivery.
  - ◦Language development, derived from the ASQ-3 subscale communication
- Child growth through questionnaires of height, weight, and head circumference at 12 and 24 months after delivery.
- Child symptoms of asthma, rhinitis, and eczema through a modified version of the ISAAC Questionnaire 24 months after delivery.
- Questionnaire on the duration of (exclusive) breastfeeding at 12 months after delivery.
- Predictive value of serum biomarkers (PlGF, sFlt) on the fetal outcome and fetal and placental size.

In RCT, participants and a control group from the cohort the following outcomes will be analyzed at 24 months after delivery:

- Resource use through a modified version of the iPCQ and iMCQ at 24 months after delivery in the RCT and a matched control group from the cohort
- Weight and height, BMI, head circumference, and blood pressure

#### Other outcomes

Long-term (10-year) cognitive, behavioral, and motor developmental and general health outcomes of the participants and/or of their infants (among others with respectively WISC, ICBCL, movement-ABC, and pediatric check-up) are planned to be evaluated. For these plans, additional funding needs to be obtained. By then, ethical board approval will be obtained in a separate amendment. Permission to approach the participants and/or their children for follow-up research will be asked via the initial informed consent.

Furthermore, the residual material of the maternal blood samples after initial analyses is planned to be used for additional fundamental research on biomedical diagnostic tools such as placental markers, RNA-markers, methylation studies, (related) protein expressions studies for example longitudinal PlGF protein levels, or plasma RNA-markers (such as CSH1, GH1, ADAM12) and placental histological characteristics following the Amsterdam consensus criteria [24].

#### Participant timeline {13}

**Fig. 1:**
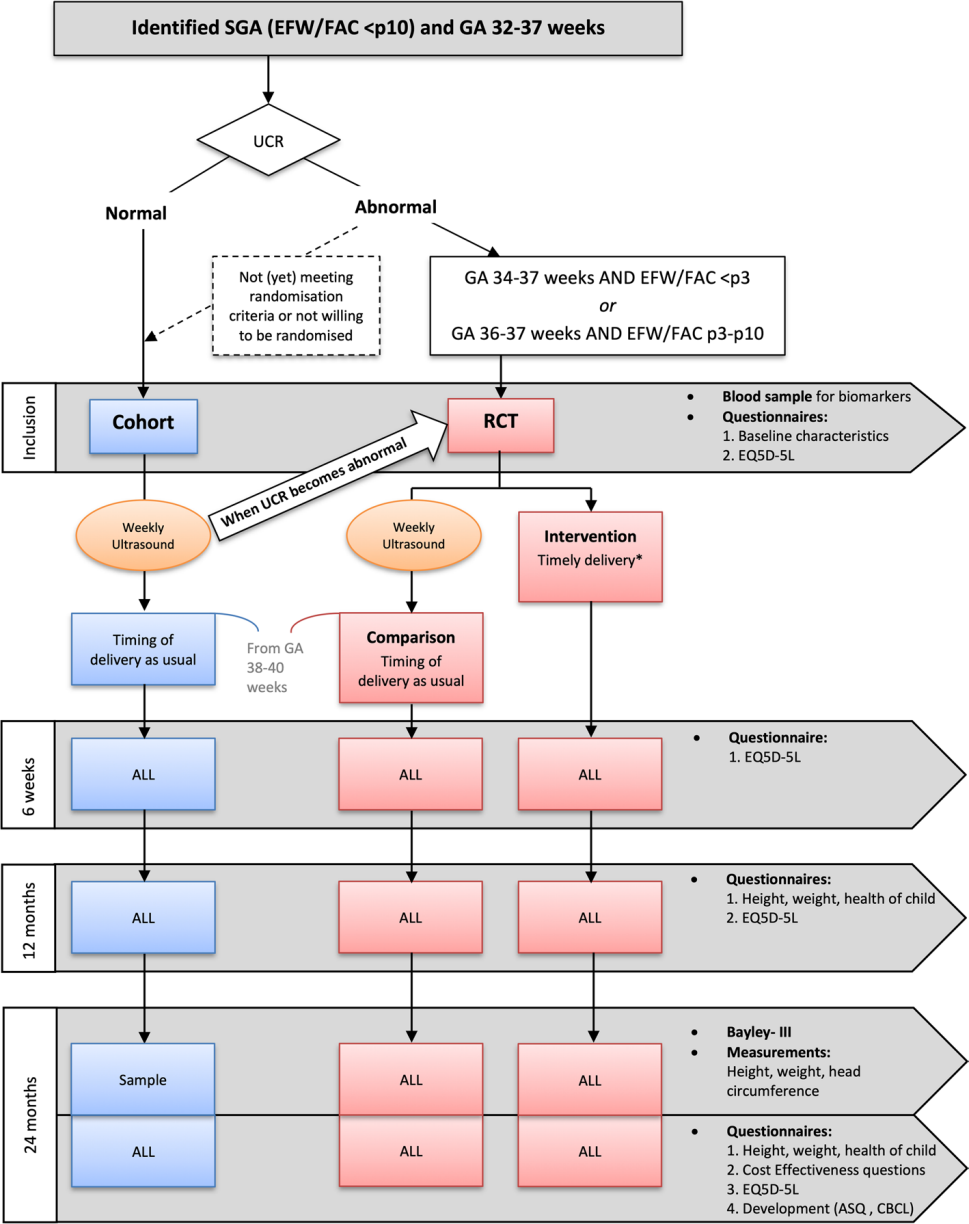
Schematic overview

Participation takes 2 years from the moment of inclusion until the last follow-up. A schematic overview is shown in Fig. 1.

#### Sample size {14}

The primary outcome is a clinically relevant difference of 7 points on the Bayley-III scale [25]. With a standard deviation of 15 points in both treatment groups, an alpha of 0.05 and a desired power of 80% for a two-sample *t-*test, 74 patients need to be recruited in each arm of the nested RCT. Accounting for a 20% loss to follow-up of randomized fetuses, 185 women will be needed for the RCT in total. Assuming a 20% incidence of abnormal UCR within the cohort of SGA fetuses and accounting for an inclusion rate in the trial of 60% we calculate to need a cohort of 1542 patients. Despite the fact that we have a history of very low rates of loss to follow-up in our RCTs, we calculate 20% loss to follow-up of randomized fetuses because of the long interval (2 years) before assessment for the primary outcomes.

#### Recruitment {15}

Patients meeting the in- and exclusion criteria will be informed about the study primarily by treating physician and secondarily by the local research midwife/nurse. The patients will also be provided with written information about the study. Furthermore, an animation video can be shown or referred to with a QR-code, to support the oral and written information. Each eligible patient must be informed that participation in the study is voluntary and that withdrawal of consent will not affect her right to the most appropriate medical treatment or affect the doctor relationship.

### Assignment of interventions: allocation

#### Sequence generation {16a}

The allocation sequence is computer-generated using the online randomization service of Castor EDC (Castor Electronic Data Capture. 2019 [online] available at https://castoredc.com.). Randomization will be done using variable block sizes of 4 and 6, and will be stratified by gestational age (dichotomous; before or after 36 weeks) and participating hospital.

#### Concealment mechanism {16b}

The allocation sequence is computer-generated using Castor EDC (Castor Electronic Data Capture, Ciwit B.N.) and thereby unknown until the intervention is assigned.

#### Implementation {16c}

Members of the local study teams are able to access the randomization service 24 h/day. After entering the needed variables (gestational age and treating center) the patient is randomized using Castor EDC.

### Assignment of interventions: blinding

#### Who will be blinded {17a}

Due to the nature of the intervention, blinding of the intervention is not applicable. Outcome assessors and data analysts are blinded.

#### Procedure for unblinding if needed {17b}

Not applicable—the design is open label with only outcome assessors being blinded so unblinding will not occur.

### Data collection and management

#### Plans for assessment and collection of outcomes {18a}

Data collection forms can be found on www.zorgevaluatienederland.nl/DRIGITAT. Trained and authorized members of the local study team will collect and enter clinical trial data. Follow-up data is collected through online questionnaires with build-inn validation checks to minimize data entry errors. The neurodevelopmental (primary) outcome data is collected and assessed by a trained developmental psychologist. Trial data is assessed by the monitoring and quality assurance board of the NVOG consortium.

#### Plans to promote participant retention and complete follow-up {18b}

In collaboration with the “Patient Journey App” an application has been developed for DRIGITAT study candidates and participants to install on their phones. This application educates patients on topics concerning small for gestational age pregnancies, fetal growth restriction as well as the DRIGITAT study. The information in the app is adjusted and updated according to the personal situation of the patient (gestational age, candidate/participant). It aims to improve the participation rate and participant retention by providing information on a regular basis and sending reminders for questionnaires.

Participants can refuse the study intervention at any time for any reason if they wish to do so. Unless they refuse to allow further data collection, such participants will continue to be followed up and will be analyzed in the group to which they were originally allocated. Participants who decline follow-up will have no further trial data collected. Any results collected up to the point at which they decline follow-up will be analyzed.

When a woman is eligible for the RCT but is not willing to be randomized for the timing of the delivery, she remains—or can be included—in the observational cohort. Additionally, these patients will be asked to complete the same follow-up as the RCT participants, as mentioned in section 13.

#### Data management {19}

Data will be collected in a web-based registry (Castor EDC) by the NVOG Consortium clinical trials unit. The computer will randomly assign a unique numeric code for every participant that bears no relation to initials or date of birth. Data handling will be done with coded data, with the key (code to personal information linkage) only available to the local investigator and the research nurse working in the local center. Persons who have access to the data include investigators, research staff, monitoring and quality assurance personnel, and the data safety monitoring board (DSMB). Data will be preserved for the duration of 15 years as laid down by Dutch statute. The handling of personal data complies with the Dutch General Data Protection Regulation (AVG).

#### Confidentiality {27}

Personal data will only be available to the local study team, personal data will always be pseudonymized for the analyses. Anonymized trial data will be shared with other researchers—on request—to enable international prospective meta-analyses.

#### Plans for collection, laboratory evaluation, and storage of biological specimens for genetic or molecular analysis in this trial/future use {33}

Maternal blood samples (serum and plasma) are taken from all participants and stored for later biomarker analysis at the moment of inclusion. Blood sampling will be repeated once a cohort participant becomes an RCT participant and the previous sample was taken more than 2 weeks earlier. Biomarker analysis of these samples is performed centrally in batch after study closure. When consent is given (see “Additional consent provisions for collection and use of participant data and biological specimens {26b}” section), residual material of this blood is stored in a biobank for 50 years for future use.

## Statistical methods

### Statistical methods for primary and secondary outcomes {20a}

Principal analyses will be performed on an intention-to-treat basis. The primary analysis will compare mean Bayley-III scores at 2 years and test the difference using a linear regression model controlling for the stratification factor. If women with suspected FGR (UCR > 0.8 or CPR < 1.25) allocated to the intervention group (timely delivery) have toddlers with better Bayley scores at 2 years, it will be concluded that timely delivery is beneficial based on UCR classification.

A sample of children born in the cohort will also be invited for Bailey-III testing and other outcomes assessed at 2 years of age. A sampling of patients from the cohort will be done against the index date of the trial patient.

Secondary endpoints will be analyzed as relative risks or differences in means with 95% confidence intervals. The chi-square tests and *t*-tests will be used for parametric dichotomous and continuous outcomes respectively. The Mann-Whitney tests with a median difference in medians (Hodges-Lehmann estimate) will be used for non-parametric continuous outcomes.

Data on pregnancy and short-term outcomes are expected only to be missing if patients withdraw consent. Long-term outcomes are anticipated to be missing in up to 15% of patients. The primary outcome will be assessed on a complete case analysis, with sensitivity analysis using multiple imputation.

An alpha of 0.05 is considered for statistical significance.

Data from the cohort study will be used as a comparator group to evaluate the course of all pregnancies, irrespective of UCR/CPR value, and inform whether different UCR/CPR thresholds or combination with other (serum) markers should be considered.

Statistical analyses will be conducted using the latest version of SPSS (SPSS Chicago, IL, USA).

### Interim analyses {21b}

A safety interim review is planned after the pregnancy outcomes of 80 RCT patients have become available without the use of inferential statistics. Hence, statistical stopping rules are not applied and correction for alpha spending is not required. Only the members of the DSMB and an independent statistician who conducts the interim review will have access to the data and reports. Given that the efficacy endpoint cannot be determined before the age of 2 years, interim analysis for efficacy is not feasible and will not be conducted.

### Methods for additional analyses (e.g., subgroup analyses) {20b}

#### Biomarker analyses

There will be subgroup analysis and biomarker analysis to evaluate the effect of early delivery on the abovementioned outcome measures in subgroups defined by (a) an abnormal or normal serum level of placenta growth factor (PlGF), (b) gestational age categories, (c) other patient characteristics available at baseline such as gestational age and estimated fetal weight, and (d) the different components of UCR (MCA, UmbA). Given the chance of spurious findings with multiple testing in a limited sample size, these analyses will be primarily hypothesis-generating and significance will be determined after Bonferroni correction.

#### Cost-effectivenesss analysis (CEA)

A trial-based cost-effectiveness analysis (CEA) will be performed, based on empirical data obtained in the RCT and a matched subgroup of the cohort. The CEA will be performed from the healthcare and societal perspective, using costs generated both within and outside of the healthcare sector. The time horizon of data collection for the economic evaluation will be 24 months, corresponding to the follow-up time and covering the neonatal care period and possible events following the neonatal period. As the intervention will mainly have a large impact on cost-effectiveness by a lifelong increase in quality-adjusted life years (QALYs) and saving of costs through improved neurological development, as measured by the Bailey-III score, we will estimate this by linking this improvement to use of healthcare resources and utility of these infants in later life by using medical literature [26, 27]. Similarly, women who have undergone a cesarean section may experience disutilities and additional costs in later life due to repeat cesarean sections. These healthcare effects and costs will also be estimated, but as with the infants, cannot be estimated directly from this trial.

The volume of health care consumption will be measured using an adapted version of the iMCQ, which the participants of the RCT and a matched subgroup of the cohort are asked to complete 24 months after delivery. This questionnaire will measure healthcare utilization for both in-hospital as well as out-of-hospital medical expenses, which include but are not limited to: medication, days in the hospital, outpatient visits after discharge, visits to the general practitioner, psychological guidance, and use of alternative medicine. Unit costs will be derived from tariffs as described in the “Zorginstituut Nederland Kostenhandleiding.” Medication costs will be derived from the “Zorginstituut Nederland Medicijnkosten” [28, 29]. If dosages are lacking in the iMCQ, standard dosages will be derived from the “Zorginstituut Nederland Farmacotherapeutisch Kompas” (WHO DDDs). In the case of uncertainty about the type of medication used by an individual, the most likely type of medication will be used. The iPCQ will be used to derive losses in productivity for the calculation of cost-effectiveness from a societal perspective. Travel costs per individual will be calculated using average travel distances and standard tariffs from the “Zorginstituut Nederland Kostenhandleiding” in combination with the number of visits to a specific medical facility as denoted by the participants in the iMCQ.

Health-related quality of life will be measured using the EQ5D-5L Questionnaire provided to women who have entered the trial at baseline, 6 weeks, 12 months, and 24 months after delivery. In the case of missing data on either costs or effects, these will be imputed by using multiple imputation techniques. The prevalence of any relevant outcomes in neonates will be extracted from CRF registration forms and electronic health records at the end of the trial. The utility scores related to these outcomes will be derived from relevant scientific literature for the calculation of cost-effectiveness. After completion of data collection, the incremental cost-effectiveness ratio (ICER) will be calculated by dividing the difference in costs between usual care and the intervention by the difference in QALYs between usual care and intervention. ICERs will be calculated for mothers and children separately and combined to analyze the potential differences in cost-effectiveness. Sensitivity analysis will be performed on parameters, which are expected to have the largest uncertainty. Bootstrapping will be used to determine the uncertainty surrounding the ICERs. The results from the bootstrap analysis will be used for plotting a cost-effectiveness acceptability curve to demonstrate the probability that the intervention strategy will be cost-effective compared to current practice when using a range of cost-effectiveness thresholds. Cost-effectiveness planes will be created to graphically represent the results from the bootstrap analysis.

#### Budget impact analysis (BIA)

A budget impact analysis (BIA) will be performed alongside the RCT to estimate the financial consequences of UCR/CPR-based delivery in women with a fetus suspected of FGR. If our study reveals that this strategy care has favorable cost-effectiveness, or is cost-saving while not reducing health outcomes, compared to standard care of intensive monitoring, the BIA will estimate the financial consequences of changing the standard care strategy from usual care to inducing delivery from gestational age more than 36 weeks in women with fetuses identified as mild SGA (EFW/FAC p3–p10) and more than 34 weeks (EFW/FAC below p3). Budget impact concerning the mother will not be very large, with an annual number of ~ 4350 patients. However, it should be considered that this is a dominant strategy, and the largest impact will be on the cost savings for these newborns in later life. Estimates for cost savings presented in the anticipated cost-effectiveness are very conservative as for the infants clinically improved Bayley-III score, corresponding to improved neurologic development, will require less resource use concerning, e.g., motor therapy [26, 29]. While the incidence of more severe neurodevelopmental morbidities, such as cerebral palsy, will be low, their health care burden is expected to be very large, partially due to their life span. The aim of the BIA is to study the costs of different scenarios for the nationwide introduction of UCR/CPR-based delivery in women with SGA fetuses. The BIA will have a simple design, consisting of a linear extrapolation of evidence collected in this project. The BIA will be performed with a time horizon of 5 years and split into results for all 5 years to demonstrate whether there turn-on-investments improve over time. BIA results will be reported separately for each year within the time horizon, and indexation will be applied. Discounting of results will not be necessary according to BIA guidelines that will be followed throughout our BIA study [30].

#### Methods in analysis to handle protocol non-adherence and any statistical methods to handle missing data {20c}

Patients who withdraw from the study will remain in their treatment group for the intent-to-treat analysis. Every effort will be made to obtain complete information on each patient randomized. The only reason for not obtaining complete information is that the patient was lost to follow-up or that she withdraws consent to access her medical chart after delivery. Once a woman has been randomized, even if she refuses the study intervention for any reason, follow-up will be continued including planned visits and maternal and fetal surveillance. Her data will be considered as per intention-to-treat in the final analysis. Imputation of outcome data will not be applied.

#### Plans to give access to the full protocol, participant-level data, and statistical code {31c}

Manuscripts resulting from this trial will be submitted to open access journals dedicated to this topic, or conventional journals providing Open Access as an option for individual publications. Original data will be made available upon request for re-use in future studies after the publication of the results.

### Oversight and monitoring

#### Composition of the coordinating center and trial steering committee {5d}

The sponsor in the coordinating center has established a working group overseeing the trial and its procedures. Procedural management is in the hands of the NVOG Consortium clinical trials unit.

#### Composition of the data monitoring committee, its role, and reporting structure {21a}

The sponsor has established a Data Safety Monitoring Board (DSMB) and will at least consist of a statistician, an epidemiologist, a gynecologist-perinatologist, and a neonatologist-pediatrician. The DSMB will meet as planned in the DSMB charter to review any unexpected adverse events. The DSMB will meet at least twice: shortly after the trial has started and to discuss the preplanned interim safety report. The DSMB has the right to review any data (unblended) that may have an impact on the trial. The DSMB can advise to terminate the study prematurely in case an interim analysis shows clear benefit or harm of either one of the interventions (timely delivery or expectant management) or due to external evidence.

The advice(s) of the DSMB will be presented to the sponsor of the study, the principal investigator, and the clinical trial unit. Should the sponsor decide not to fully implement the advice of the DSMB, the sponsor will present the advice to the reviewing ethical board approval, including a note to substantiate why (part of) the advice of the DSMB will not be followed. The most recent DSMB-charter can be found on www.zorgevaluatienederland.nl/DRIGITAT.

#### Adverse event reporting and harms {22}

Whether or not considered related to the study intervention, any serious adverse event (SAE) that occurs in patients in the RCT from the time of signed consent through 30 days after hospital discharge must be reported within 24 h to one of the individual(s) listed on the contact form. Guidelines on SAE reporting in the DRIGITAT study can be found on www.zorgevaluatienederland.nl/DRIGITAT.

#### Frequency and plans for auditing trial conduct {23}

Monitoring will be performed in compliance with Good Clinical Practice (GCP) and other rules and regulations in order to achieve high-quality research and secure patient safety. Monitoring will be coordinated by the NVOG Consortium and will be conducted by a qualified independent monitor. Based on the site-specific monitoring plan of the NVOG Consortium, monitoring visits to each participating site will be performed every year. The independent monitor will have access to the data and source documents of the trial to review the quality of the participating sites. For more detailed information, see the latest monitor plan at the website (www.zorgevaluatienederland.nl/DRIGITAT).

#### Plans for communicating important protocol amendments to relevant parties (e.g., trial participants, ethical committees) {25}

All substantial amendments will be notified and approved by the Dutch ethical research committee (METC) and the competent authority. Important protocol modifications will be communicated to the participating sites.

#### Dissemination plans {31a}

After completing the trial and data analysis, the results of the trial will be published as soon as possible in an international journal on obstetrics. We aim to publish within 1 year after completing the trial. If the results indicate that our national protocol for FGR needs adjustment an amendment will be planned

## Discussion

Intervention trials in pregnancies complicated with FGR as defined according to the consensus definition are scarce due to its diagnostic complexity. The current study will evaluate whether SGA fetuses suffering from placental insufficiency as measured by an abnormal UCR benefit from early delivery.

A strength of this study is the large cohort of small fetuses without Doppler abnormalities to compare the results of the RCT with and to determine whether these fetuses are rightly considered to be constitutionally small and healthy.

A limitation of this study is the exclusion of AGA fetuses that may suffer van FGR. The current clinical practice (e.g., local protocols) and the still prevailing idea that AGA fetuses are not at risk from the consequences of FGR made it unfeasible to investigate brainsparing in AGA fetuses in the same manner. This still remains a major challenge to overcome in the future.

### Operational or practical issues

Due to the differences in local FGR protocols, we expect that the management of patients slightly differ between different hospitals influencing the outcomes of this study. We try to overcome this by providing clear guidelines on the management and diagnostics used in the study population. In addition, some of the possible differences in standard care can be traced through data from the CRFs.

This trial is based on both Doppler and biometric ultrasound measurements, with all its known limitations in inter- and intra-observer variability and accuracy. Although suboptimal, it reflects the current clinical practice and remains the gold standard in detecting FGR in the absence of superior alternatives. To minimize the inter-observer variability in Doppler measurements, we provided e-learnings.

### Trial status

Recruitment began on 24 September 2018 in the coordinating center. Recruitment is planned to be completed in the second half of 2023.

## Data Availability

The datasets generated during and/or analyzed during the current study are available from the corresponding author on reasonable request

## Abbreviations

ABR: ABR form, General Assessment and Registration form, is the application form that is required for submission to the accredited Ethics Committee (In Dutch, ABR = Algemene Beoordeling en Registratie)
AE: Adverse event
AEDF: Absent end-diastolic flow
AFI: Amniotic fluid index
AGA: Appropriate for gestational age
ASQ-3: Ages & Stages Questionnaire, Third Edition
AVG: Algemene Verordening Gegevensbescherming
BIA: Budget impact analysis
BMI: Body mass index
CA: Competent authority
CBCL/1.5-5: Child Behavior Checklist for ages 1.5–5
CCMO: Central Committee on Research Involving Human Subjects; in Dutch: Centrale Commissie Mensgebonden Onderzoek
CP: Cerebral palsy
CPR: Cerebroplacental ratio
CRF: Case report form
CS: Cesarean section
CSH1: Chorionic somatomammotropin hormone 1
CTG: Cardiotocography
CV: Curriculum vitae
DSMB: Data Safety Monitoring Board
EFW: Estimated fetal weight
EQ5D-5L: European Quality of life 5-Dimension 5-Level
FAC: Fetal abdominal circumference
FGR: Fetal growth restriction
FL: Femur length
GCP: Good Clinical Practice
GH1: Growth hormone 1
GMFCS: Gross Motor Function Classification Scale
HC: Head circumference
HELLP: Hemolysis elevated liver enzyme low platelets
IB: Investigator’s brochure
IC: Informed consent
iMCQ: Medical Consumption Questionnaire
IMP: Investigational medicinal product
IMPD: Investigational Medicinal Product Dossier
iPCQ: Productivity Cost Questionnaire
IQ: Intelligence quotient
ISAAC: International Study of Asthma and Allergies in Children - Questionnaire
MCA: Middle cerebral artery
MDI: Mental Developmental Index
METC: Medical research ethics committee (MREC); in Dutch: medisch ethische toetsing commissie (METC)
NICU: Neonatal intensive care unit
Movement-ABC: Movement Assessment Battery for Children
PDI: Psychomotor Developmental Index
PI: Pulsatility index
PlGF: Placental growth factor
QALY: Quality-adjusted life year
QR-code: Quick response code
RCT: Randomized controlled trial
REDF: Reversed end-diastolic flow
(S)AE: (Serious) adverse event
SDP: Single deepest pocket
sFlt: Soluble fms-like tyrosine kinase
SES: Socioeconomic status
SGA: Small for gestational age
UCR: Umbilicocerebral ratio
UmbA: Umbilical artery
UtA: Uterine artery
WMO: Medical Research Involving Human Subjects Act (in Dutch: Wet Medisch-wetenschappelijk Onderzoek met Mensen)
WPPSI: Wechsler Preschool and Primary Scale of Intelligence
